# White Wine Intake Reduces Mitochondrial Complex I-Derived ROS in the Male Rat Heart

**DOI:** 10.3390/antiox15091199

**Published:** 2026-09-19

**Authors:** Jeronim Matijevic, Marko Grahovac, Marin Mornar, Ivana Novak, Marko Ljubkovic, Mladen Boban, Jasna Marinovic

**Affiliations:** 1 Department of Basic and Clinical Pharmacology, University of Split School of Medicine, 21000 Split, Croatia; jeronim.matijevic@kbsplit.hr (J.M.);; 2 Department of Immunology and Medical Genetics, University of Split School of Medicine, 21000 Split, Croatia; 3 Department of Physiology, University of Split School of Medicine, 21000 Split, Croatia

**Keywords:** white wine, myocardial mitochondria, reactive oxygen species, ischemia/reperfusion, transcriptomics

## Abstract

Epidemiological studies suggest that, despite the adverse effects of excessive alcohol intake, low-to-moderate levels of wine consumption have been associated with favorable cardiovascular outcomes. While the biological effects of polyphenol-rich red wine have been extensively studied, less is known about the impact of white wine on myocardial cellular function and the underlying mechanisms. In this study, young male Sprague-Dawley rats were given ad libitum access to white wine or water for four weeks, after which cardiac mitochondrial function, cardiomyocyte susceptibility to oxidative stress, and cardiac transcriptomic profiles were assessed using respirometry, ROS measurements, in vitro oxidative stress assay, and RNA sequencing. White wine consumption did not significantly alter mitochondrial respiratory capacity, expression of antioxidant enzymes or cardiomyocyte survival following oxidative challenge. However, mitochondrial ROS production at respiratory complex I was significantly reduced, particularly during reverse electron transport mimicking ischemia/reperfusion. Transcriptomic analysis revealed modest changes, with no genes meeting the false discovery rate threshold. Analyses based on nominal significance revealed changes associated with mitochondrial electron transfer, protein quality control, apoptosis, and inflammatory signaling. In conclusion, four-week white wine consumption was associated with reduced mitochondrial ROS without impairing cardiac bioenergetics, accompanied by subtle transcriptional responses.

## 1. Introduction

Epidemiological studies have reported associations between light-to-moderate wine consumption and lower cardiovascular risk [1]. A landmark study published in 1992 reported lower mortality from ischemic heart disease in France compared with other European countries, despite a high prevalence of major risk factors such as saturated fat intake, smoking, and sedentary lifestyle [2]. This observation, later termed the “French paradox,” was largely attributed to wine consumption, particularly red wine. Subsequent studies have shown that moderate wine intake may influence biological processes relevant to cardiovascular disease, including lipoprotein profile, thrombosis, inflammation, and oxidative balance [1,3]. These effects have been mainly attributed to polyphenols, a heterogeneous group of bioactive compounds present in wine, whose composition and concentration depend on grape variety, environmental conditions, and winemaking procedures [3,4].

White wine is typically produced either from white grape varieties or from red grape varieties processed with minimal skin contact. Because phenolic compounds are concentrated in the skins and seeds, reduced skin maceration generally results in lower total polyphenol content than in red wine [5]. Nevertheless, white wines still include various phenolic and non-phenolic bioactive compounds, and experimental studies in rats have reported reduced infarct size, improved post-infarction healing, and increased survival after sustained white wine intake [6,7,8,9,10,11]. Compared with red wine, however, mechanistic studies addressing the effects of white wine remain limited.

The cardiovascular effects of wine and alcohol are complex and strongly influenced by the amount and pattern of consumption, with heavy and binge drinking associated with worse cardiovascular outcomes compared to mild-to-moderate sustained consumption [1,12]. In the heart, excessive alcohol intake is associated with adverse outcomes including alcoholic cardiomyopathy, chronic heart failure, and cardiac arrhythmias [13]. Proposed mechanisms underlying these detrimental effects include increased oxidative stress, induction of apoptosis, and impairment of mitochondrial metabolism and contractile function [14].

A substantial body of experimental evidence implicates mitochondrial dysfunction and altered redox regulation in pathological myocardial remodeling and heart failure [15]. Moreover, modulation of mitochondrial function and ROS generation is also implicated in mechanisms that limit myocardial injury [16]. Given their central role in energy production, redox regulation and cell survival, mitochondria are therefore involved in mechanisms of pathological cardiac remodeling, as well as those of cardioprotection [15,16]. However, whether sustained white wine intake influences mitochondrial function remains poorly understood.

Considering the complex and dose-dependent effects of white wine, as well as the relative paucity of mechanistic data, the present study investigated the effects of sustained white wine intake on myocardial mitochondrial function, cardiomyocyte susceptibility to oxidative stress, and cardiac gene expression in healthy rats. We hypothesized that sustained voluntary white wine intake would be associated with measurable changes in myocardial mitochondrial redox homeostasis and transcriptional responses in healthy rat hearts.

## 2. Materials and Methods

The study was performed at the University of Split School of Medicine. All experimental protocols and procedures were approved by the Institutional Ethics Committee and by the Directorate of Veterinary and Food Safety of the Ministry of Agriculture of the Republic of Croatia (No: 525-10/1338-21-5).

### 2.1. Study Design

Sample size was determined a priori using power analysis assuming a two-tailed test, α = 0.05, power = 0.8, and an expected effect size derived from preliminary data. Sprague-Dawley male rats (approximately 4 weeks old and weighing ~150 g at the beginning of the experiment) were randomly assigned to two groups: the Wine group (n = 20), which drank white wine (Grasevina/Welschriesling—a white grape variety from eastern Croatia, produced by Krauthaker winery, with 13.0% alcohol), and the Control group (n = 15), which drank only water. Only male Sprague–Dawley rats were included in the study to reduce biological variability related to estrous-dependent hormonal influences [17] and to maintain consistency with our previously established experimental model of white wine consumption. Rats were housed in separate cages under standardized conditions (22–24 °C, 12 h light/dark cycle) and had continuous access to a standard pellet diet. The Wine group had access to wine 24 h/day and access to water for six hours daily. The control group had unrestricted access to water. Liquid intake was assessed daily, and food intake and body weight were monitored weekly.

After 28 days, hearts were harvested for downstream analyses. Because the assessed endpoints required mutually incompatible tissue preparation protocols, not all analyses could be performed in the same individual animals and each experiment was performed on a defined subset of animals, as specified in the figure legends.

### 2.2. Cardiomyocyte Isolation

Rats were anesthetized with ketamine (90 mg/kg) and xylazine (8 mg/kg) injected in the right hamstring muscle. The hearts were excised, mounted on a Langendorff apparatus and retrogradely perfused with heparinized Joklik medium containing collagenase type II (Invitrogen, Carlsberg, CA, USA), protease XIV, and bovine serum albumin (both from Sigma-Aldrich, Merck, Darmstadt, Germany). After enzymatic digestion, isolated cardiomyocytes were obtained as described previously [18] and stored in Tyrode solution (in mM: 132 NaCl, 10 HEPES, 5 glucose, 5 KCl, 1 CaCl_2_, and 1.2 MgCl_2_, pH 7.4) at room temperature until use.

### 2.3. Assessment of Cardiomyocyte Tolerance to Acute Oxidative Stress

A suspension of isolated cardiomyocytes (1 mL) was transferred to a perfusion chamber mounted on the stage of an inverted microscope (Olympus CKX31, Tokyo, Japan). After allowing the cells to settle and adhere to the chamber bottom for approximately 10 min, cardiomyocytes were continuously perfused with glucose-free Tyrode solution. Viable rod-shaped cardiomyocytes were identified and counted under light microscopy. In each experiment, approximately 350 myocytes were counted, and the counting period was kept constant across all experiments.

Oxidative stress was induced by perfusion with glucose-free Tyrode solution supplemented with 200 µM H_2_O_2_ and 100 µM FeSO_4_·7H_2_O for 17 min. This combination generates highly reactive hydroxyl radicals (·OH) via the Fenton reaction. The exposure duration of 17 min was determined in preliminary experiments to result in approximately 50% cell damage. Following oxidative stress, cardiomyocytes were washed with glucose-free Tyrode solution, and the number of surviving viable cells was counted again. Cell survival was expressed as the percentage of viable cardiomyocytes remaining after oxidative stress relative to baseline.

In addition, cardiomyocyte surface area was quantified from light microscopy images using ImageJ 1.52q software (NIH, Bethesda, MD, USA).

### 2.4. Flow Cytometric Assessment of Cardiomyocyte Antioxidant and Stress-Response Proteins

Fixed cardiac single-cell suspensions were permeabilized with 0.01% Triton X-100 for 10 min. Following washing, cells were incubated with primary antibodies for 30 min. Antibodies against heat shock protein 70 (HSP70), glutathione peroxidase (GPx), and catalase (CAT) (Santa Cruz Biotechnology, Dallas, TX, USA) were directly conjugated to Alexa Fluor 488 (AF488). The primary antibody against NAD(P)H quinone dehydrogenase 1 (NQO1) (Santa Cruz Biotechnology) was unconjugated and was therefore detected using an Alexa Fluor 647 (AF647)-conjugated secondary antibody (BioLegend, San Diego, CA, USA). An anti-cardiac troponin antibody (Abcam, Cambridge, UK) was used to identify the cardiomyocyte population.

Samples were analyzed using a BD Accuri C6 flow cytometer (BD Biosciences, San Jose, CA, USA). Unstained and secondary-antibody-only controls were used to establish background fluorescence, with a 2% background threshold used to define positive and negative populations. Cardiomyocytes identified by cardiac troponin staining formed a distinct population based on forward- and side-scatter characteristics (FSC/SSC). Based on the established fluorescence threshold, the percentage of cardiomyocytes positive for each protein was determined. Individual values were subsequently normalized to the mean value of the Control group, which was set to 1, and are presented as fold of Control. Flow cytometry data were analyzed using FCS Express 7 software (De Novo Software, Pasadena, CA, USA).

### 2.5. Mitochondrial Respiration

Tissue oxygen consumption rate and hydrogen peroxide (H_2_O_2_) production were measured simultaneously using a Clark-type oxygen electrode equipped with a fluorescence detection module (O2k-FluoRespirometry system, Oroboros Instruments, Innsbruck, Austria). Measurements were performed on freshly excised left ventricular tissue, which was immediately washed and cleaned in ice-cold biopsy preservation solution (BIOPS, in mM: 7.23 K_2_EGTA, 2.77 CaK_2_EGTA, 50 K-methanesulfonate, 6.56 MgCl_2_, 5.77 Na_2_ATP, 15 Na_2_Phosphocreatine, 20 imidazole, 20 taurine, 0.5 dithiothreitol; pH adjusted to 7.1 at 0 °C with KOH). The wet weight of each tissue sample was carefully determined.

BIOPS was subsequently washed out and replaced with mitochondrial respiration medium (MiR05, in mM: 110 sucrose, 20 HEPES, 20 taurine, 10 KH_2_PO4, 0.5 EGTA, 60 lactobionic acid, 3 MgCl_2_; with 1 g/L bovine serum albumin, pH adjusted to 7.1 at 30 °C with KOH) supplemented with 5 mM pyruvate and 0.5 mM malate. The tissue was homogenized using a semi-automated mechanical grinder (PBI Shredder SG3 system, Pressure Biosciences, Canton, MA, USA), and the resulting homogenate was immediately transferred into the respirometry chambers. All measurements were performed at 30 °C, with oxygen concentration maintained between 200 and 220 µM to avoid oxygen-dependent effects on mitochondrial respiration and ROS production.

Mitochondrial respiratory states were evaluated by sequential addition of substrates, uncouplers, and inhibitors. In the presence of the NADH-generating substrates pyruvate (5 mM), malate (0.5 mM), and glutamate (5 mM), addition of ADP (2.5 mM) stimulated oxidative phosphorylation supported by complex I (OxPhos CI). Subsequent addition of succinate (10 mM) yielded maximal oxidative phosphorylation supported by complexes I and II (OxPhos CI+CII). Maximal electron transfer system capacity (ETS) was then determined by stepwise titration of the uncoupler carbonyl cyanide p-trifluoromethoxyphenylhydrazone (FCCP). Finally, inhibition of complex I with rotenone (0.5 μM) revealed the remaining uncoupled complex II-supported respiration (ETS CII).

### 2.6. Mitochondrial Production of Superoxide

Superoxide production was assessed indirectly by measuring the rate of H_2_O_2_ formation, as superoxide is rapidly converted to H_2_O_2_ by endogenous and exogenously added superoxide dismutase (SOD). H_2_O_2_ production was quantified using the Amplex UltraRed detection system. Myocardial homogenates were incubated with 2.5 μM Amplex UltraRed (Thermo Fisher Scientific, Waltham, MA, USA), 1 U/mL horseradish peroxidase (HRP) and 5 U/mL SOD. Fluorescence of the resorufin-like reaction product, generated in a 1:1 ratio with H_2_O_2_, was recorded at 587 nm (excitation at 525 nm). Background fluorescence, measured in the absence of tissue, was subtracted in every recording, and the fluorescent signal was calibrated using freshly prepared H_2_O_2_ standards (0.1 μM).

ROS production was assessed under experimental conditions designed to selectively stimulate three canonical modes of mitochondrial ROS generation: complex I-linked reverse electron transport (RET), complex I-linked forward electron transport (FET), and maximal oxidative phosphorylation (OxPhos CI+CII). For the RET and FET protocols, ROS production was quantified as the increase in H_2_O_2_ production rate elicited by the specific intervention establishing the respective experimental condition. In contrast, ROS production during OxPhos CI+CII was determined as the steady-state H_2_O_2_ production rate measured under phosphorylating conditions after background subtraction.

RET at complex I occurs under conditions of high protonmotive force (Δp) and a highly reduced ubiquinone pool, which together drive electron backflow toward complex I. To establish these conditions, homogenates were first incubated with the NADH-generating substrates pyruvate, malate, and glutamate until a stable baseline fluorescence signal was achieved. Succinate was then added at a saturating concentration to reduce the ubiquinone pool, in the absence of exogenous ADP and in the presence of oligomycin (100 nM) to preserve Δp by inhibiting ATP synthase. The increase in H_2_O_2_ production rate following succinate addition was quantified as RET-associated ROS production. This increase was subsequently abolished by the protonophore FCCP, confirming its dependence on Δp [19].

FET at complex I refers to forward electron flow from NADH-linked substrates to ubiquinone. In these experiments, homogenates were incubated with pyruvate, malate, and glutamate in the absence of ADP until a stable fluorescence signal was achieved. ROS production during FET was then stimulated by addition of rotenone (0.5 μM), an inhibitor of the complex I ubiquinone-binding (I_Q_) site. The increase in H_2_O_2_ production rate following rotenone addition was quantified as complex I-associated ROS production under FET conditions [20].

ROS production during maximal oxidative phosphorylation was assessed in the presence of pyruvate, malate, glutamate, succinate, and saturating ADP. After stabilization of the fluorescence signal, the background-subtracted steady-state H_2_O_2_ production rate was taken as ROS production under OxPhos CI+CII conditions.

### 2.7. RNA Isolation

Snap-frozen heart tissue was thawed overnight at −20 °C in RNAlater-ICE Frozen Tissue Transition Solution (Ambion/Thermo Fisher Scientific, Waltham, MA, USA). Total RNA was isolated using TRIzol Reagent (Invitrogen/Thermo Fisher Scientific, Waltham, MA, USA) according to the manufacturer’s instructions. Purified RNA was dissolved in DEPC-treated water. RNA integrity was assessed using the RNA Nano 6000 Assay Kit of the Bioanalyzer 2100 system (Agilent Technologies, Santa Clara, CA, USA).

### 2.8. RNA Sequencing and Bioinformatics Analysis

RNA sequencing libraries were prepared using the NEB Next Ultra RNA Library Prep Kit for Illumina, and sequencing was performed by Novogene (Beijing, China). Each sample was sequenced to a depth of approximately 40 million reads using a 150 bp paired-end strategy on the Illumina NovaSeq platform (Illumina, Inc., San Diego, CA, USA). The quality of raw reads was assessed and filtered. Reads containing adapter contamination, reads with >10% ambiguous nucleotides (N), and reads in which >50% of bases had a Phred quality score < 5 were removed. Filtered reads were aligned to the rat reference genome using HISAT2. Transcript assembly was performed using StringTie (v1.3.3b) [21], and gene-level read counts were obtained using FeatureCounts (v1.5.0-p3). Gene expression levels were estimated as FPKM (Fragments Per Kilobase of transcript per Million mapped reads), accounting for gene length and sequencing depth. Differential expression analysis between Control and Wine groups was performed using the DESeq2 R package (v1.20.0), based on raw read counts. Genes with an adjusted *p*-value (*p*-adj) ≤ 0.05 were considered significantly differentially expressed. To assess the biological relevance of expression changes, Gene Set Enrichment Analysis (GSEA) based on Gene Ontology (GO) and KEGG gene sets was performed using the GSEA software v3.0 (Broad Institute Cambridge, MA, USA).

### 2.9. Statistical Analysis

Data normality was assessed using the Shapiro–Wilk test. Comparisons between two groups were performed using an unpaired Student’s *t*-test. Statistical significance was defined as *p* < 0.05. Unless otherwise stated (e.g., in tables), data are presented as mean ± SD. All statistical analyses, except for RNA-seq data, were performed using GraphPad Prism 10.

## 3. Results

### 3.1. Wine Intake, Baseline Physiological Characteristics and Cardiomyocyte Stress Tolerance

As shown in Table 1, rats in the Wine group consumed approximately 12 mL of wine per day. Based on the ethanol content of the white wine (13% *v*/*v*), the density of ethanol (0.789 g/mL), and an average body weight of 225 g during the study period, this intake corresponded to an ethanol dose of approximately 5.47 g/kg/day [22].

**Figure 1:**
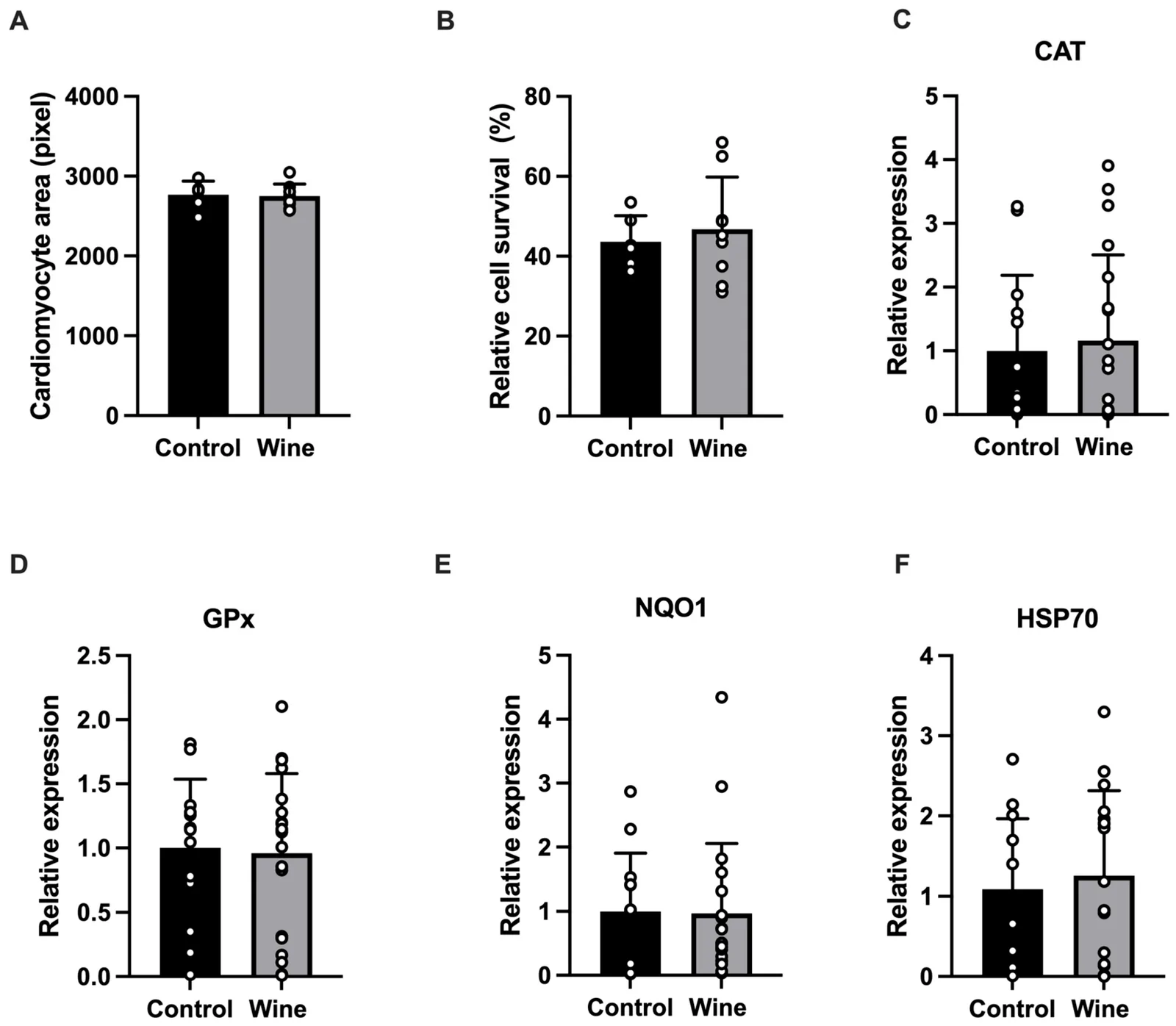
Effects of wine intake on cardiomyocyte size, sensitivity to oxidative stress, and expression of antioxidant and stress-response proteins. Data are presented as mean ± SD for the Control (black) and Wine (grey) groups. (**A**) Average area of isolated cardiomyocytes, expressed as pixel number. (**B**) Percentage of isolated cardiomyocytes surviving in vitro exposure to oxidative stress. (**C**–**F**) Flow cytometric assessment of cardiomyocyte protein expression of catalase (CAT), glutathione peroxidase (GPx), NAD(P)H quinone dehydrogenase 1 (NQO1), and heat shock protein 70 (HSP70), respectively. Protein expression was normalized to the mean value of the Control group, which was set to 1. Each point represents one animal. For (**A**,**B**), n = 6 in the Control group and n = 9 in the Wine group; for (**C**–**F**), n = 13 in the Control group and n = 19 in the Wine group.

During the four-week intervention, both groups gained approximately 150 g of body weight, despite lower food intake in the Wine group (Table 1). This likely reflects partial caloric substitution by ethanol-derived calories, as body weight gain remained similar between groups. Morphometric analysis of isolated ventricular cardiomyocytes revealed no differences in myocyte area between groups (Figure 1A). Similarly, the percentage of cardiomyocytes surviving in vitro exposure to oxidative stress did not differ between experimental groups (Figure 1B). Flow-cytometric analysis of cardiomyocyte expression of proteins involved in antioxidant and cellular stress responses revealed no significant differences between the Control and Wine groups in the expression of CAT, GPx, NQO1, or HSP70 (Figure 1C–F).

### 3.2. Cardiac Mitochondrial Respiration and ROS Production

**Figure 2:**
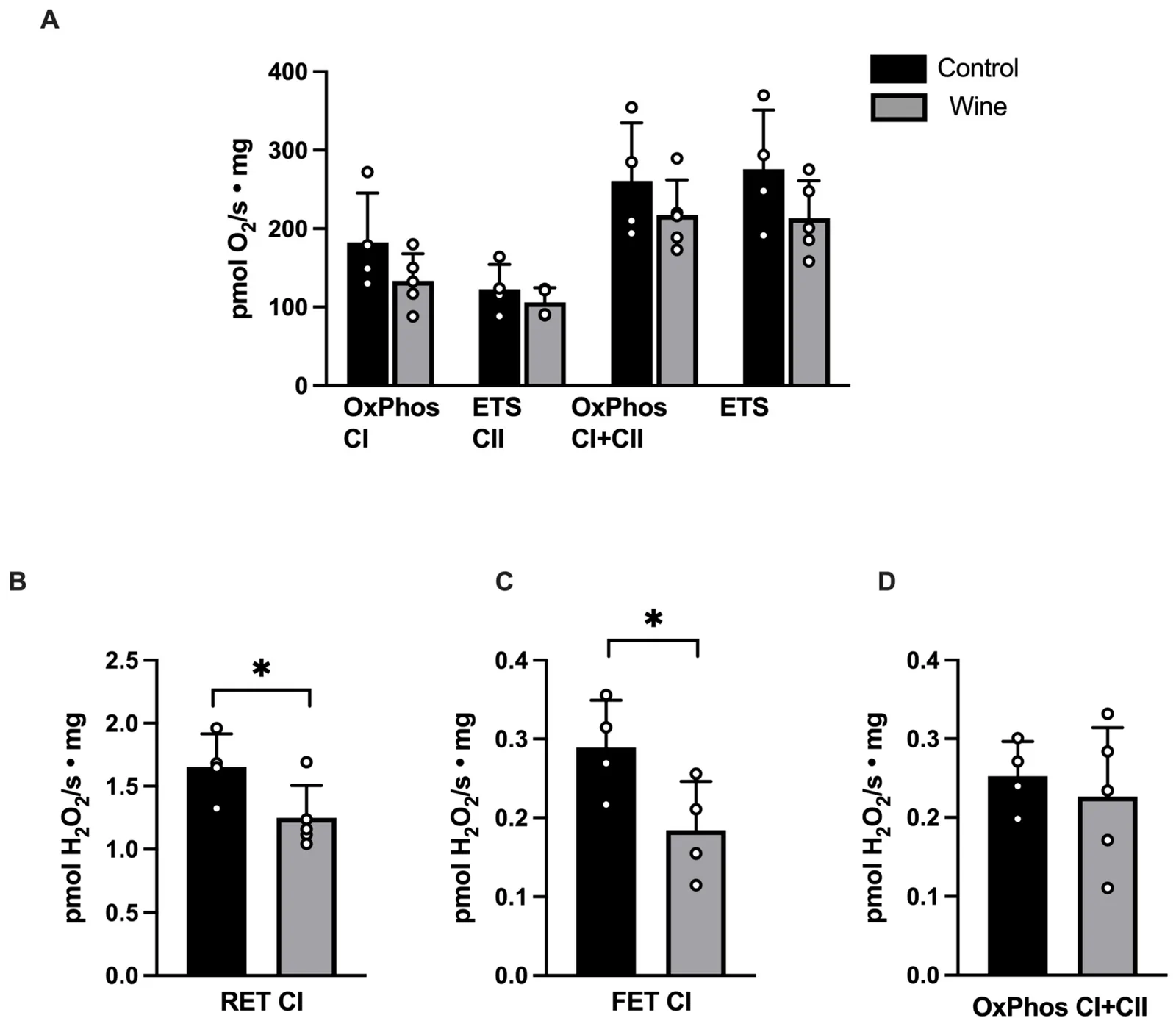
Effects of wine intake on mitochondrial respiration and superoxide production. (**A**) Mitochondrial oxygen consumption mediated by complex I (OxPhos CI), complex II (ETS CII), maximal oxidative phosphorylation capacity in the presence of all substrates (OxPhos CI+CII), and maximal electron transfer system capacity (ETS) assessed after uncoupling with FCCP. (**B**–**D**) Mitochondrial superoxide production was assessed indirectly by measuring the rate of H_2_O_2_ formation following superoxide dismutation. Superoxide production was measured (**B**) under conditions mimicking early reperfusion, characterized by reverse electron transport (RET) at complex I; (**C**) during forward electron transport (FET) at complex I induced by rotenone-mediated inhibition of the ubiquinone-binding site in the presence of NADH-linked substrates; and (**D**) under regular OxPhos conditions (OxPhos CI+CII). Data are presented as mean ± SD. n = 4 in Control group and n = 5 in Wine group. * *p* < 0.05, Wine versus Control group.

Comparison of mitochondrial respiratory function revealed no significant differences between the Wine and Control groups in any of the assessed respiratory states, including OxPhos supported by complex I (OxPhos CI), maximal OxPhos supported by complexes I and II (OxPhos CI+CII), maximal electron transfer system capacity (ETS), and uncoupled complex II-supported respiration (ETS CII) (Figure 2A).

Mitochondrial ROS production was first assessed under experimental conditions mimicking early reperfusion, in which RET at complex I is considered a major source of superoxide generation [23]. As shown in Figure 2B, the increase in H_2_O_2_ production rate induced by the establishment of RET conditions was significantly lower in cardiac mitochondria from the Wine group than in Controls. To further assess complex I-associated ROS generation, FET-associated ROS production was quantified as the increase in H_2_O_2_ production rate following rotenone-induced inhibition of the I_Q_ site. As shown in Figure 2C, this response was likewise significantly attenuated in the Wine group. In contrast, the steady-state net H_2_O_2_ release measured during OxPhos CI+CII did not differ significantly between groups (Figure 2D).

### 3.3. Myocardial Gene Expression Changes Following White Wine Consumption

**Figure 3:**
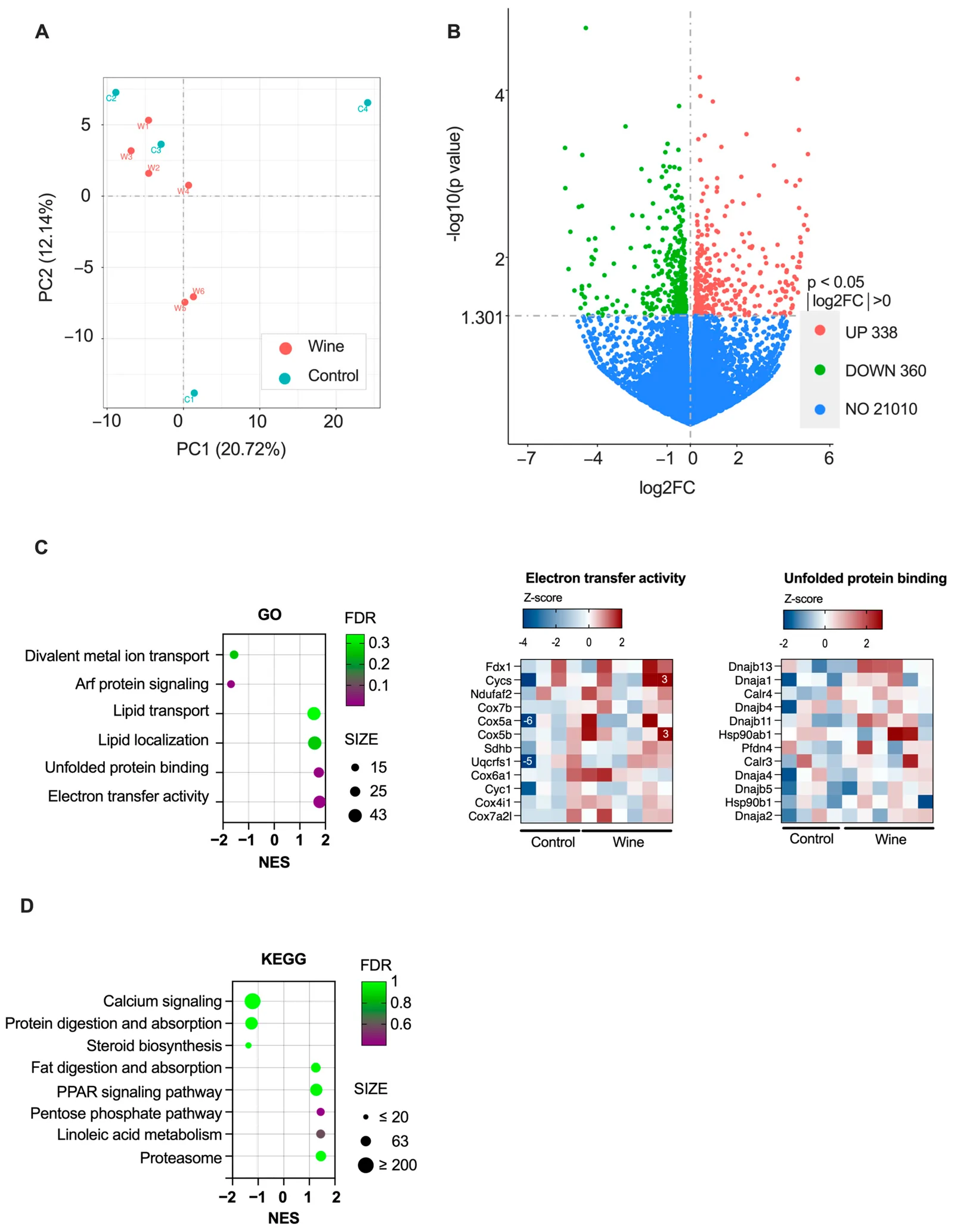
Effect of wine intake on myocardial gene expression. (**A**) Principal component analysis (PCA) of myocardial transcriptomes from Control (n = 4) and Wine groups (n = 6). (**B**) Volcano plot showing differential gene expression between Wine and Control groups based on nominal significance (*p* < 0.05, “NO” means not differentially expressed). (**C**) Left panel, Gene Ontology (GO) terms enriched in the Wine group as identified by Gene Set Enrichment Analysis (GSEA). NES, normalized enrichment score; FDR, false discovery rate. Right panels, heatmaps illustrating the increased expression of specific gene sets in electron transfer activity and unfolded protein binding GO terms. Numbers on the heatmap denote values outside the legend range. (**D**) KEGG pathways analyzed by GSEA; no pathways reached statistical significance. log2FC, log2 fold change; NES, normalized enrichment score; FDR, false discovery rate.

RNA sequencing analysis revealed modest changes in myocardial gene expression following four weeks of wine intake. Principal component analysis did not show clear separation between groups (Figure 3A). Consistent with this observation, no individual genes met the threshold for differential expression after correction for multiple comparisons (*p*-adj > 0.05 for all genes).

Analysis based on nominal significance identified 698 genes with *p* < 0.05, of which 360 were upregulated and 338 downregulated in the Wine group compared with Controls (Figure 3B). To identify functional patterns associated with these transcriptional changes, downstream enrichment analyses were performed using all genes meeting the nominal significance criterion (*p* < 0.05). GSEA of GO terms revealed enrichment of gene sets related to electron transfer activity and unfolded protein binding in the Wine group (Figure 3C). In contrast, GO terms associated with Arf protein signal transduction, participating in membrane trafficking, cytoskeletal organization, vesicular transport and lipid metabolism [24], were downregulated. No KEGG pathways were significantly enriched in response to wine consumption (Figure 3D).

Genes with the largest log2 fold change (log2FC) are listed in Table 2. Among the most upregulated transcripts was gamma-glutamyltransferase 1 (*Ggt1*), a commonly used biomarker of alcohol exposure and liver dysfunction, consistent with systemic exposure to ethanol. In contrast, the most downregulated transcript was *Soat2*, which encodes sterol O-acyltransferase 2, an enzyme involved in cholesterol metabolism.

## 4. Discussion

The current study demonstrates that four-week intake of white wine in healthy rats attenuates myocardial mitochondrial ROS production under conditions in which complex I is a major contributor, particularly during reverse electron transport (RET) and rotenone-sensitive forward electron transport (FET). These effects occurred without significant changes in mitochondrial respiratory capacity or cardiomyocyte sensitivity to exogenous oxidative stress. In parallel, RNA sequencing revealed subtle transcriptional changes associated with mitochondrial electron transfer, protein quality control, apoptosis, and inflammatory signaling.

Over the years, extensive evidence has documented both potentially beneficial effects of wine and the toxic effects of alcohol on various organs, including the cardiovascular system. To further characterize the cardiac effects of white wine under controlled conditions, we exposed young, healthy rats to ad libitum white wine for four weeks. This sub-chronic intervention allowed us to study early responses to wine consumption, prior to functional changes associated with aging. Based on body surface area scaling, the ethanol exposure achieved in the present study corresponds to approximately 0.88 g/kg/day in humans, or roughly 60 g ethanol/day for a 70 kg adult [22]. Although this exceeds most definitions of moderate human alcohol intake, similar exposure ranges are commonly used in voluntary ethanol-drinking rodent models [25].

Previous studies have shown that both ethanol and non-alcoholic white wine extracts may attenuate ischemia/reperfusion injury [7,26]. We therefore investigated whether sustained white wine intake influences cardiomyocyte sensitivity to oxidative stress, an important contributor to ischemia/reperfusion injury. However, in vitro oxidative stress induced similar levels of cell injury in both groups, suggesting that enhanced intrinsic cardiomyocyte resistance to oxidative stress is unlikely to account for the beneficial effects of white wine previously observed in experimental ischemia/reperfusion models [8,9,10]. In parallel, flow cytometry analysis revealed no differences between groups in cardiomyocyte expression of antioxidant and cellular stress-response proteins CAT, GPx, NQO1, and HSP70.

Mitochondria are both a major source of ROS and a target of ROS-induced injury during ischemia/reperfusion [23,27]. ROS bursts generated upon reperfusion damage mitochondrial components, impair ATP production, and trigger mitochondrial permeability transition, ultimately leading to cardiomyocyte death, tissue injury, and inflammation [28]. Beyond ischemia/reperfusion, mitochondrial oxidative stress is also implicated in ethanol-induced organ damage [14]. Most studies examining the mitochondrial effects of ethanol have focused on the liver, where acute exposure increases superoxide production at complexes I and III [29]. In cardiac models, chronic ethanol exposure has been associated with impaired mitochondrial respiration, increased ROS production, and contractile dysfunction in a dose-dependent manner [30,31].

In the present study, sustained white wine intake did not significantly alter mitochondrial respiratory capacity but was associated with reduced ROS production linked to complex I during both forward electron transport (FET) and reverse electron transport (RET). This observation contrasts with the predominantly detrimental mitochondrial effects reported following chronic ethanol exposure and may reflect the influence of non-ethanolic wine constituents. The wine used in the present study was a commercially available Grasevina/Welschriesling previously characterized by our group, containing 13.0% ethanol and approximately 305 mg gallic acid equivalents (GAE)/L of total phenolics [32]. However, because the present study did not include an ethanol-only control group, it cannot distinguish whether the observed reduction in ROS is attributable to ethanol, non-alcoholic wine constituents, or interactions between these components. Previous experimental studies, including our own work using the same model of white wine consumption, have suggested that non-alcoholic constituents of wine may contribute to biological effects beyond those attributable to ethanol alone [11]. In that study, white wine, but not an equivalent amount of ethanol, significantly improved survival following experimentally induced myocardial infarction in rats [11].

Complex I (NADH: ubiquinone oxidoreductase) is the primary entry point for electrons into the mitochondrial respiratory chain and plays a central role in ATP production. At physiological levels, ROS generated at complex I participate in redox signaling processes involved in calcium handling, excitation-contraction coupling and metabolic adaptations [33]. However, under pathological conditions, complex I can become a major source of excessive ROS, contributing to oxidative damage and cardiomyocyte injury, with RET recognized as a key driver of ROS generation during early reperfusion in vivo [23,34]. RET-derived ROS depends on multiple factors including complex I activity, the redox state of the ubiquinone pool, and the magnitude of the protonmotive force [19,35]. Accordingly, the reduction in ROS observed in the present study may reflect alterations in respiratory chain protein function, mitochondrial energetics, membrane potential, redox balance, or upstream regulatory pathways.

In parallel with the functional analyses, we examined whether sustained white wine intake is associated with changes in myocardial gene expression. Comparison of myocardial transcriptomes between Control and Wine groups by PCA revealed only subtle differences after four weeks of wine intake. Consistent with this, no individual transcripts remained significantly different after correction for multiple testing, suggesting that the intervention did not induce major transcriptional remodeling in the healthy rat myocardium.

Despite the absence of FDR-significant genes, analysis based on nominal significance (*p* < 0.05) identified changes in transcripts related to oxidative stress regulation, mitochondrial function, protein quality control, apoptosis, and inflammatory signaling. Among the nominally upregulated transcripts was *Ggt1*, a clinically used biomarker of alcohol exposure encoding an enzyme involved in glutathione metabolism and cellular defense against oxidative stress [36]. Also, expression of the mitochondrial uncoupling protein 2 transcript (*Ucp2*; log2FC = 0.76, *p* = 0.002) was increased, accompanied by enrichment of gene sets related to electron transfer activity. Ucp2 has been implicated in modulation of mitochondrial ROS production [37]. Nominal transcriptional changes were also observed in genes involved in apoptotic signaling, including reduced expression of *Casp3* (log2FC = −0.52, *p* < 0.05) and increased expression of the anti-apoptotic Fas apoptosis inhibitory molecule (*FAIM*; log2FC = 2.2, *p* < 0.05).

In addition, several nominally regulated transcripts were associated with inflammatory and lipid-handling pathways relevant to cardiovascular disease, including *Soat2*, *Chrna7*, and *Clcf1* (cardiotrophin-like cytokine 1; log2FC = −1.17, *p* = 0.004) [38,39,40,41]. *Soat2* encodes an endoplasmic reticulum enzyme involved in cholesterol esterification and lipid droplet formation [38]. *Chrna7* signaling modulates vascular inflammation and contributes to the development of atherosclerosis [40], while *CLCF1* promotes macrophage transition to foam cells, a key step in atherogenesis [41].

Our study has several limitations. First, different experimental endpoints were assessed in separate subsets of animals because of incompatible tissue preparation requirements. Consequently, mitochondrial, cellular, and transcriptomic findings could not be directly correlated within the same individual animal. Second, the exclusive use of male rats limits extrapolation of the findings to females, particularly in light of known sex differences in cardiac mitochondrial physiology and myocardial susceptibility to ischemia/reperfusion injury [17,42]. In addition, although reduced ROS generation was observed under complex I-linked conditions (RET and FET), the mechanistic basis of this effect cannot be determined from the present data. Specifically, we did not directly assess complex I enzymatic activity or mitochondrial membrane potential, which may influence complex I-associated ROS production. Also, as we did not include an experimental group receiving an equivalent amount of ethanol, the observed effects cannot be attributed specifically to ethanol, non-alcoholic wine constituents, or interactions between these components. Finally, transcriptomic analysis revealed modest changes in myocardial gene expression, with no genes reaching FDR significance after correction for multiple testing, likely reflecting the subtle transcriptional impact of four weeks of white wine intake in healthy myocardium.

## 5. Conclusions

In conclusion, sustained white wine intake in healthy rats was associated with reduced mitochondrial ROS generation at complex I, without significant alterations in mitochondrial respiratory capacity or cardiomyocyte resistance to exogenous oxidative stress. Transcriptomic analysis revealed subtle changes in genes related to mitochondrial electron transfer, protein quality control, apoptosis, and inflammatory signaling. Together, these findings suggest selective modulation of mitochondrial redox homeostasis under conditions relevant to ischemia/reperfusion, although the functional implications of these observations remain to be established.

## Figures and Tables

**Table 1:** Water, wine and food intake, and body weight gain in Control (n = 15) and Wine (n = 20) drinking groups. * *p* < 0.05.

Group	Water Intake (mL)	Wine Intake (mL)	Food Intake (g)	Weight Gain (g)
**Control**	34.9 ± 5.7	0	620.7 ± 114	152.3 ± 15.7
**Wine**	12.9 ± 3.5 *	11.9 ± 4.6	526.7 ± 19 *	151.2 ± 26.2

**Table 2:** Genes exhibiting the largest log2 fold change (log2FC) with *p* < 0.05. Positive log2FC values indicate upregulated expression, while negative values indicate downregulated expression in the Wine group compared with the Control group.

Gene Name	Log2FC	*p*	*p*-Adj	Gene Description
**Up**				
***Hist1h2bb***	4.98	0.003	0.98	histone cluster 1, H2bb
***Spsb2***	4.89	0.004	0.99	splA/ryanodine receptor domain and SOCS box containing 2
***Cysltr2***	4.75	0.009	0.99	cysteinyl leukotriene receptor 2
***Ceacam18***	4.71	0.001	0.8	carcinoembryonic antigen-related cell adhesion molecule 18
***Cnih3***	4.71	0.01	0.99	cornichon family AMPA receptor auxiliary protein 3
***Oprd1***	4.68	0.01	0.99	opioid receptor, delta 1
***Akr1d1***	4.60	0.01	0.99	aldo-keto reductase family 1, member D1
***Ggt1***	4.48	0.02	0.99	gamma-glutamyltransferase 1
***Gpr25***	4.45	0.02	0.99	G protein-coupled receptor 25
***Rnf225***	4.41	0.009	0.99	ring finger protein 225
**Down**				
***Soat2***	−5.16	0.004	0.99	sterol O-acyltransferase 2
***Kcng3***	−4.99	0.02	0.99	potassium channel, voltage gated modifier subfamily G, member 3
***Tcerg1l***	−4.66	0.02	0.99	transcription elongation regulator 1-like
***Sult4a1***	−4.66	0.02	0.99	sulfotransferase family 4A, member 1
***Atp6v0a4***	−4.64	0.0005	0.71	ATPase, H+ transporting, lysosomal V0 subunit A4
***Chga***	−4.57	0.03	0.99	chromogranin A
***Fank1***	−4.54	0.006	0.99	fibronectin type 3 and ankyrin repeat domains 1
***Chrna7***	−4.38	0.006	0.99	cholinergic receptor, nicotinic, alpha 7
***Mylk4***	−4.24	0.01	0.99	myosin light chain kinase family. member 4
***Klk15***	−4.15	0.01	0.99	kallikrein-related peptidase 15

## Data Availability

The data underlying this article will be shared upon reasonable request to the corresponding author. Raw sequencing data are openly available at NCBI SRA Database (Bioproject ID PRJNA1521705; http://www.ncbi.nlm.nih.gov/bioproject/1521705, accessed on 1 September 2026).
